# Vigorous exertion, regular exercise training, and the risk of sudden cardiac death due to myocardial infarction in Swedish men

**DOI:** 10.1016/j.ijcrp.2026.200588

**Published:** 2026-02-03

**Authors:** Simon Vikström, Patrik Wennberg, Lars Johansson, Marcus Lind, Elin Chorell, Johan Sommar, Jonas Andersson

**Affiliations:** a Department of Public Health and Clinical Medicine, Skellefteå Research Unit, Umeå University, Sweden; b Department of Public Health and Clinical Medicine, Family Medicine, Umeå University, Sweden; c Department of Public Health and Clinical Medicine, Section of Medicine, Umeå University, Sweden; d Department of Public Health and Clinical Medicine, Section of Sustainable Health, Umeå University, Sweden

**Keywords:** Sudden cardiac death, Myocardial infarction, Physical activity, Mortality, Risk factor

## Abstract

**Background:**

Although physical activity is associated with cardiovascular health benefits and reduced all-cause mortality, vigorous exertion is also recognized as a trigger for sudden cardiac death (SCD). This study investigated vigorous exertion as a trigger for SCD resulting from myocardial infarction (MI) and the potential modifying effect of habitual vigorous exercise training among Swedish men who subsequently experienced SCD due to MI.

**Methods:**

This prospective nested case-crossover study was performed within the Västerbotten Intervention Programme cohort from 1985 to 2006 and included male participants who later experienced SCD caused by MI. The risk of SCD during and within 30 min of vigorous exertion was compared with the risk during periods of non-exertion. Participants were categorized into three groups according to baseline frequency of habitual vigorous exercise training to assess effect modification.

**Results:**

We included 192 men with SCD caused by MI, with a mean time from screening to event of 6.5 years. A majority of cases reported physical inactivity, with 161 cases reporting no exercise or <1 exercise event per week. 24 men suffered SCD in relation to vigorous exertion, yielding a relative risk of 43.6 (95% CI: 27.1-70.3) compared to non-exertion. The highest relative risk (107.7 [95% CI: 63.4-182.9]) was found among physically inactive men and was mitigated by a higher frequency of habitual exercise training at baseline.

**Conclusion:**

Among Swedish men who experienced SCD caused by MI, vigorous exertion was associated with a transiently increased risk, which was mitigated by higher levels of habitual vigorous exercise training.

## Graphical abstract

**Figure:**
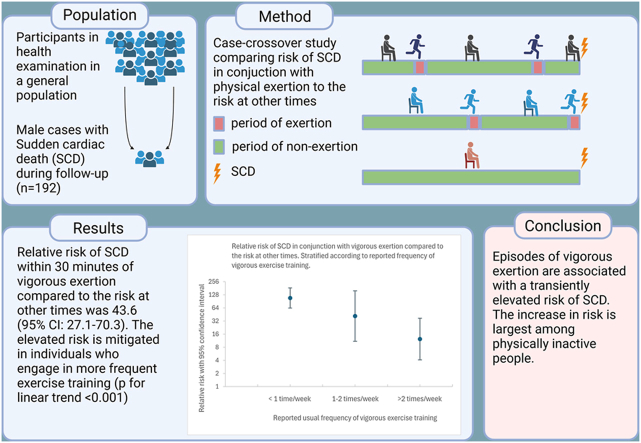

## 1 Introduction

Sudden cardiac death (SCD) accounts for approximately 50% of total cardiovascular mortality [1]. In many cases, SCD is the first symptom of ischaemic heart disease (IHD), meaning that a significant proportion of SCD victims have not had the opportunity to access the advances in secondary preventive treatment for IHD that have emerged over the past few decades. To reduce mortality, it is important to improve primary prevention by identifying risk factors and triggers for SCD. Regular moderate to vigorous physical activity has consistently been shown to benefit cardiovascular health and reduce all-cause mortality [2]. On the other hand, it has been shown that vigorous exertion can act as a trigger for both myocardial infarction (MI) and SCD, though regular vigorous exercise training may diminish that risk [[3], [4], [5], [6], [7]]. Previous studies of SCD triggered by exertion were performed in select populations, such as male physicians [5] and female nurses [6]. To the best of our knowledge, no large prospective study has been conducted on a general population. Given that IHD is the predominant cause of SCD in individuals aged ≥35 years, it is pertinent to investigate whether vigorous physical activity can serve as a precipitating factor for SCD resulting from MI. The aim of the present study was to study associations between vigorous exertion and SCD. We hypothesized that episodes of vigorous exertion are associated with an elevated risk of SCD caused by MI, and that habitual vigorous exercise training attenuates that risk.

## 2 Methods

### 2.1 Study design

We employed a prospective nested case-crossover design to investigate potential triggering factors that occur before the SCD. In this design, each individual serves as their own control, comparing the risk of SCD during vigorous exertion and during a short period after vigorous exertion to the risk of SCD at other times. This approach is suitable when the exposure is intermittent with an immediate and transient effect on risk [8,9], such as exercise training.

The study protocol conforms to the ethical guidelines of the 1975 Declaration of Helsinki. The protocol was approved by the Regional Ethics Review Board of Umeå (no. 2011-87-31M). Informed consent was obtained from all subjects involved in the study.

### 2.2 Study population

The study population was derived from the Västerbotten Intervention Programme (VIP) [10] with cases identified using the MI event registration within the northern Sweden monitoring trends and determinants in cardiovascular disease (MONICA) study [11]. This framework allows us to examine SCD based on prospective data from a representative general population. Baseline characteristics for the population are presented in Table 1. VIP was launched in 1985 with the aim to reduce morbidity and mortality from cardiovascular disease and diabetes, and it has been integrated into primary health care in Västerbotten since the 1990s. VIP includes a health examination, an extensive questionnaire, standard blood analysis, and health dialogue with a trained nurse. Inhabitants in Västerbotten County are invited to the VIP at 40, 50, and 60 years old. The MI event registration is based on reports from general practitioners, hospital discharge records, autopsy reports, and death certificates. By identifying cases of MI with SCD in the event registry among patients who had also participated in VIP between 1985 and 2006, we were able to include individuals who suffered SCD during that time, including those who died out-of-hospital and cases with no attempted resuscitation. We defined SCD as death within 24 h of MI. An event was classified as triggered by vigorous exertion if death – or the onset of MI symptoms that resulted in death within 24 h – occurred during or within 30 min after vigorous exertion. MI was classified according to WHO criteria. Definite MI was defined as either: serial ECG progression according to Minnesota codes; cardiac enzyme levels exceeding twice the upper limit of normal in combination with compatible symptoms and ECG findings; or autopsy findings showing recent MI or coronary thrombosis. Cases not meeting criteria for definite MI were classified as possible MI if there was a history of IHD but no autopsy was performed; if the autopsy report showed signs of chronic occlusive coronary disease or old infarction; or if there were typical or atypical symptoms before death and no evidence of another cause of death was identified. All cases with definite or possible MI in which death occurred within 24 h of symptom onset were classified as SCD due to MI and were eligible for inclusion. SCD was classified by a trained nurse and, when needed, in dialogue with an experienced cardiologist. Methods for classification are described in detail elsewhere [12].

**Table 1:** Baseline characteristics.

	SCD related to vigorous exertion (n = 24)	SCD not related to vigorous exertion (n = 168)	Any SCD (n = 192)
Mean (SD)			
Age at screening, years	53.4 (6.4)	55.0 (6.5)	54.8 (6.5)
Age at event, years	59.6 (6.5)	61.5 (7.6)	61.3 (7.5)
Time to event, years	6.2 (3.7)	6.6 (4.2)	6.5 (4.1)
BMI, kg/m^2^	27.9 (3.7)	27.6 (3.9)	27.6 (3.9)
Total cholesterol, mmol/L	6.7 (1.5)	6.3 (1.1)	6.4 (1.1)
No. (%)			
Sex (male)	24 (100)	168 (100)	192 (100)
Frequency of vigorous exercise			
<1 time/week	17 (70.8)	144 (85.7)	161 (83.9)
1-2 times/week	3 (12.5)	8 (4.8)	11 (5.7)
>2 times/week	4 (16.7)	16 (9.5)	20 (10.4)
Low level of education	11 (45.8)	99 (58.9)	110 (57.3)
Diabetes	1 (4.2)	31 (18.5)	32 (16.7)
Hypertension	15 (62.5)	98 (58.3)	113 (58.9)
Active smoking	9 (37.5)	73 (43.5)	82 (42.7)
Prior MI	3 (12.5)	33 (19.6)	36 (18.8)
Out of hospital SCD	22 (91.7)	127 (75.6)	149 (77.6)
			

			
			
			
			
			

			

			
			
			
			
			
			
			
			
			

SCD: sudden cardiac death, BMI: body mass index, MI: myocardial infarction.

### 2.3 Baseline variables

Baseline data were retrieved from the health examination records of the VIP. Blood pressure was measured in a supine position. Hypertension was defined as systolic blood pressure ≥140 mmHg, diastolic blood pressure ≥90 mmHg, or the use of antihypertensive drugs. The body mass index (BMI) was calculated as weight in kilograms divided by height in meters squared. A bench top analyser (Reflotron) was used to analyse total cholesterol and capillary plasma glucose. Diabetes was defined as plasma glucose ≥7.0 mmol/L, oral glucose tolerance test ≥12.2 mmol/L, self-reported diabetes on the questionnaire, or the use of anti-diabetic drugs. A low level of education was defined as ≤9 years of compulsory education. Current smoking did not include occasional smoking or former smoking. Information on prior MI was obtained from the questionnaire and medical records.

Data to estimate the frequency of habitual exercise training was derived from the question, “How often during the last 3 months have you practiced or exercised in training clothes for the purpose of improving your fitness or to feel well?” The response options were: "never," "occasionally," "1-2 times per week," "2-3 times per week," or "more than 3 times per week," which were designated numerical values of 0, 0.5, 1.5, 2.5, and 4 times per week, respectively. We also categorized the population according to the frequency of exercise. Those who reported “never” or “occasionally” were combined into the category of "less than 1 time per week." The value "1-2 times per week" was retained, and the two remaining groups were combined in the category "more than 2 times per week.” No data were available on the intensity, type of exercise, or duration of each episode, but as the question included “in training clothes,” we assumed the intensity to be at least 6 metabolic equivalents of task (MET), which is the established definition of vigorous activity. The duration of each episode of vigorous exercise training was assumed to be 30 min on average.

### 2.4 Physical activity in conjunction with the event

Information about physical activity within 1 h of the onset of symptoms was obtained from medical records and reviewed by an experienced nurse or general practitioner. Classification of physical activity intensity was based on estimated MET levels using a standard MET chart, rather than on specific activity type. All forms of physical activity were considered and categorized as asleep, sitting/light sitting activity, light activity, moderate activity, or vigorous activity, where vigorous activity was defined as ≥6 MET. Examples of activities found in the category of vigorous activity were snow shovelling, running and cross-country skiing. Unwitnessed cases were classified as missing unless there was clear evidence of what activity was performed at the time of death. For the analysis, we dichotomized the categories as vigorous activity or not vigorous activity.

### 2.5 Statistical analysis

Baseline characteristics are presented as means and standard deviations or numbers and proportions. Relative risks are presented with 95% confidence intervals.

When analysing case-crossover data, standard methods for cohorts with sparse data in each stratum are applicable. The stratifying variable is each subject. Each episode of vigorous exertion and a period of time thereafter, a “carry-over” time, was considered to be a period of exposure referred to as the “effect period.” The time interval preceding the onset of symptoms is referred to as the “hazard period.” We calculated the Mantel-Haenszel rate ratio as a measure of relative risk with control periods based on the usual frequency approach [13]. Using this method, we calculated the relative risk by comparing the observed event rate during hours of exposed person-time to the expected event rate based on the frequency of habitual exercise training reported at baseline. We chose an effect period of 1 h (30 min of vigorous exercise and 30 min carry-over) based on previous studies and the composition of our data. Exposed person time was calculated by multiplying the usual weekly frequency of exercise training by 12 (approximately 12 weeks per 3 months, 1 h effect time). Unexposed time was determined by subtracting the exposed time from the overall hours in 12 weeks. To study any modifying effect by the frequency of habitual exercise training on the relative risk, we stratified the data according to the habitual frequency of exercise training and performed a chi-squared test for linear trend.

### 2.6 Sensitivity analyses

We performed three sensitivity analyses. First, we tested the sensitivity to our choice of effect period by recalculating the relative risks using different combinations of the duration of vigorous exercise and carry-over, 30-60 min and 0-60 min, respectively. Second, we considered the habitual exercise training to encompass not only vigorous, but also moderate exertion and included moderate to vigorous exertion in conjunction with SCD as triggered cases. Finally, because VIP is an intervention programme, it is possible that a temporary change in the frequency of habitual exercise training occurred that is likely more pronounced in a limited time frame after the VIP visit. Therefore, we excluded cases with an event within 2 years from participation in the VIP and re-calculated the relative risks.

## 3 Results

We identified 398 patients who suffered SCD resulting from MI who had participated in the VIP. Of these, 73 patients had missing data regarding habitual vigorous exercise and were excluded from our analysis. We also excluded an additional 89 cases for which information about physical activity preceding death was missing. In total, 236 cases of SCD had complete data and could be included in the analysis. All patients with SCD triggered by vigorous exertion were male. Consequently, we were unable to conduct any sex-stratified analysis. As a result, we excluded all females from the study. This ultimately left 192 patients, all of whom were male, and they formed the basis of our analysis (Supplement 1). Mean time from screening to event was 6.5 years. Among all cases, 24 cases of SCD were related to vigorous exertion. Baseline characteristics are outlined in Table 1. A history of MI was present in 18.8% of cases, and 77.6% of the SCD events occurred out-of-hospital. Autopsy was performed in 60.4% of the cases. The mean age at event was 61.3 years, and 83.9% of the patients reported a physically inactive lifestyle with a frequency of habitual physical exercise of less than once per week.

We found a relative risk of 43.6 (95% CI: 27.1-70.3) for SCD during vigorous exertion and within 30 min following the activity compared to other periods. The relative risk was highest (107.7 [95% CI: 63.4-182.9]) among patients with a low frequency of habitual vigorous exercise. However, it gradually decreased as the exercise frequency increased (p for linear trend <0.001) but remained significantly elevated at 12.3 (95% CI: 4.1-36.8) in the group with the highest habitual frequency (Table 2). The sensitivity analysis yielded variations in the magnitude of the estimates, but the main result, that the risk of SCD is increased in association with episodes of vigorous exertion and is modified by habitual exercise training, remained consistent across all sensitivity analyses (Supplement 2).

**Table 2:** Relative risk of sudden cardiac death (SCD) within 30 min of vigorous exertion in men.

Frequency of habitual vigorous exercise	Total SCD events	SCD related to vigorous exertion	Relative risk (95% CI)^a^
All	192	24	43.6 (27.1-70.3)
<1 time/week	161	17	107.7 (63.4-182.9)
1-2 times/week	11	3	41.6 (11.0-157.7)
>2 times/week	20	4	12.3 (4.1-36.8)

a Compared to the risk of SCD at other times. CI denotes confidence interval.

## 4 Discussion

In this prospective case-crossover study on a male population in northern Sweden, we found a significantly increased risk of SCD associated with episodes of vigorous exertion compared with periods of non-exertion. The increase in risk was attenuated by a higher frequency of habitual vigorous exercise. Although the relative risk was lower among physically active subjects, it remained significantly elevated. Nevertheless, the absolute excess risk of SCD in conjunction with vigorous exertion has been shown to be very low [5,6] and sports-associated SCD probably represent only a small proportion of overall SCD [14,15]. Importantly, exercise training has been recognized as beneficial for cardiovascular health and is associated with a lower overall risk of SCD [16]. Exercise capacity, commonly quantified as peak oxygen uptake (VO_2_ max), is an independent predictor of both short- and long-term cardiovascular outcomes in individuals at high cardiovascular risk [17,18]. The findings of our present study should therefore be interpreted in the context of overall cardiovascular risk and functional capacity. Avoidance of vigorous physical activity due to concern about acute risk may paradoxically increase long-term cardiovascular risk by accelerating functional decline. Consequently, the public health objective should be the maintenance or improvement of cardiorespiratory fitness. Sustaining adequate levels of physical activity remains a major challenge. In this context, telemedicine and mobile health interventions have emerged as promising tools to support long-term exercise adherence and facilitate improvements in functional capacity and cardiovascular risk profiles [[19], [20], [21], [22]]. The magnitude of the relative risk in our population is larger than what has been reported in previous studies but, when studying the stratified analysis of different habitual frequencies of exercise training, the magnitudes are more consistent across the different studies. As relative risks consistently appear to be significantly higher among individuals with lower physical activity levels in similar studies, the high proportion of physically inactive persons in our dataset probably contributes to a high relative risk for the entire population. Our study provides further evidence supporting previous findings and reinforcing the notion that vigorous exertion can act as a trigger for SCD. Furthermore, it highlights the beneficial effect of regular vigorous exercise training in mitigating the transiently elevated risk, as demonstrated within a population-based cohort. These findings underscore the need for a well-balanced approach to exercise, emphasizing the benefits of physical activity for cardiovascular health, while also being aware of the transiently elevated SCD risk during vigorous exertion. Regular exercise remains a cornerstone of cardiovascular health, and these results emphasize its importance in reducing the risk of SCD.

Our primary findings remained unaltered in all sensitivity analyses. The relative risk increased when we chose a shorter hazard period and decreased with a longer hazard period. This was expected, as almost all SCD events associated with vigorous exertion occurred during the physical activity. It is important to acknowledge that the calculated relative risks are highly dependent on our assumptions regarding the duration of physical activity and carry-over time. Therefore, the magnitudes should be regarded as estimates and subject to these assumptions.

The role of physical activity in preventing SCD remains incompletely understood. It is firmly established that exercise attenuates traditional risk factors for IHD, such as blood pressure, glucose control, and lipids. Consequently, this effect should also extend to reducing the risk of SCD. However, more immediate beneficial effects that cannot be attributed to the attenuation of traditional cardiovascular risk factors have also been proposed [23,24]. These effects encompass alterations in myocardial oxygen demand, plaque vulnerability, susceptibility to malignant arrhythmias, and variations in inflammatory and haemostatic factors.

The risk of SCD associated with vigorous exertion appears to be different in males and females, with females having a lower increase in risk than males. In the all-female cohort of the “Nurses' Health Study”, the relative risk of SCD in conjunction with moderate to vigorous exertion was 2.4 (95% CI: 1.2-4.6), with very few patients suffering SCD related to physical activity [6]. This contrasts with the all-male cohort in the “Physicians’ Health Study”, in which the corresponding relative risk was 16.9 (95% CI: 10.5-27.0)^5^. In our population, less than 20% of patients with SCD were female, and none of the events were associated with vigorous exertion. Although we could not perform any sex or gender-based analysis, we can conclude that SCD is more common among males and that SCD associated with vigorous exertion is an extremely rare event among females. The differences between males and females could be attributed, in part, to the higher prevalence of IHD among males. However, it is probable that additional as yet unidentified factors also play a role, warranting further investigation.

## 5 Strengths and limitations

The prospective design of this study is an important strength because the risk of recall bias in self-reported physical activity is reduced. The case-crossover design eliminates confounding by factors that are constant within individuals over time. Approximately one-third of the SCD cases were confirmed by autopsy as being attributable to MI, enhancing the study's internal validity. Utilizing a local event register for MI and the inclusion of out-of-hospital SCD cases represent significant advantages by ensuring a more comprehensive inclusion of cases. Remarkably, more than 75% of the cases in our study pertained to out-of-hospital SCD, and a substantial portion of these cases are estimated to not have involved attempted resuscitation and, consequently, may not be accounted for in any of Sweden's national registers. Their inclusion reduced selection bias and increased the statistical power of our study.

This study also has several limitations. One such limitation is the limited number of subjects with SCD in association with vigorous exertion. The small numbers make the study vulnerable to misclassifications of SCD caused by MI. Although we used validated cases to minimize this risk, a substantial proportion of events were out-of-hospital deaths and consequently, many cases did not meet the criteria for definite MI, but were classified as possible MI. This may have led to both under- and overestimation of SCD due to MI and makes the estimated relative risk less reliable, especially when we stratify data. However, our classification approach was conservative, and any resulting misclassification is more likely to have attenuated associations rather than exaggerated them. In addition, almost 50% of the SCD cases were excluded due to missing baseline data. This reduced the statistical power and, if missing data were not random, could introduce bias into the results. In particular, cases lacking information on physical activity prior to death are problematic in this aspect, as exertion is more likely to be recorded in medical documentation than rest. Furthermore, in unwitnessed SCD, classification of physical activity preceding death was based on the circumstances in which the subject was found. Uncertain cases were classified as missing, which in turn could lead to an underestimation of the number of cases related to vigorous exertion. The extended period between the collection of baseline data to the event poses a challenge. As the study design uses self-matching, there is no need to adjust for factors that are constant over time. However, the time interval to event was 6 years, during which subjects could have altered their habitual frequency of exercise training, potentially resulting in misclassification. This is beyond our control and could introduce bias into the results, either positively or negatively. We acknowledge the possibility of additional factors that were not accounted for in our study and may have introduced confounding effects into the results. We lacked information on important clinical characteristics such as heart failure status, left ventricular ejection fraction, and the severity of any underlying coronary artery disease. These factors may influence both susceptibility to exertion-triggered SCD and an individual's capacity and willingness to engage in habitual vigorous physical activity. Differences in underlying disease burden may therefore partly explain the observed associations. As this is an observational study, causal inferences cannot be drawn. In addition, our study lacked information regarding the duration of each episode of exercise training. While we explored this in our sensitivity analysis, it did not affect the main result; however, it did influence the magnitude of the relative risk. This raises concerns about the reliability of these magnitudes. There is a risk of misclassification of the reported habitual frequency of exercise training because we had no detailed data on the intensity of the activity. It is plausible that some of the habitual exercise we assumed to be vigorous was, in fact, moderate intensity.

## 6 Conclusion

Engaging in vigorous exertion was associated with a transiently elevated risk of SCD resulting from acute MI. This elevated risk was mitigated in individuals who engaged in more frequent habitual vigorous exercise training.

## CRediT authorship contribution statement

**Simon Vikström:** Writing – original draft, Methodology, Formal analysis, Conceptualization. **Patrik Wennberg:** Writing – review & editing, Methodology, Data curation. **Lars Johansson:** Writing – review & editing, Methodology. **Marcus Lind:** Writing – review & editing, Methodology. **Elin Chorell:** Writing – review & editing, Methodology. **Johan Sommar:** Writing – review & editing, Formal analysis. **Jonas Andersson:** Writing – review & editing, Supervision, Methodology, Conceptualization.

## Funding

This study was supported by grants from 10.13039/501100002915Region Västerbotten and the Foundation for Medical Research in Skellefteå.

## Declaration of competing interest

None declared.

## Data Availability

Access to individual-level data can be provided for research purposes but is restricted by laws regarding the privacy of research participants and are therefore not made publicly available. Requests for data can be sent to the Section of Biobank and Registry Support at Umeå University (contact: info.brs@umu.se)
