# Supplementary material for: Vigorous exertion, regular exercise training, and the risk of sudden cardiac death due to myocardial infarction in Swedish men
Source: Int J Cardiol Cardiovasc Risk Prev. 2026 Feb 3;29:200588. doi: 10.1016/j.ijcrp.2026.200588 (PMC12907004; doi:10.1016/j.ijcrp.2026.200588)
Supplement: Multimedia component 1 [file mmc1.docx]

**Supplement 1


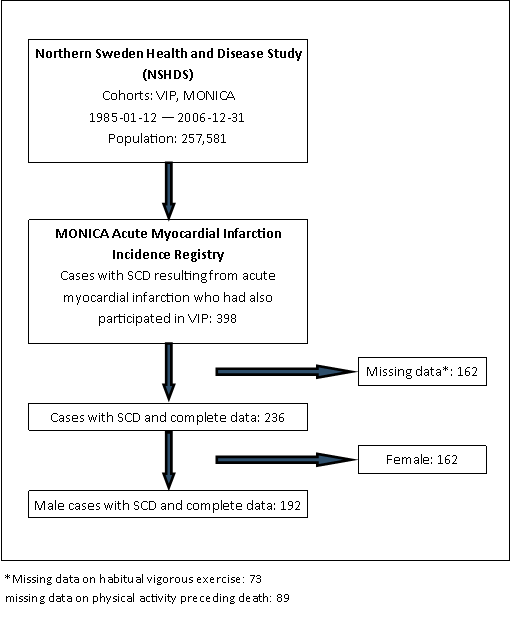


Supplement 2**

Sensitivity analysis.

1) Different combinations of duration of exercise and carry-over time. The effect time is the sum of the duration of exercise and carry-over time. In the main analysis, we assumed an episode of exercise to be 30 min with a carry-over time of 30 min, resulting in 60 min exposure to risk at each episode of vigorous exercise training.

| **Table 3. Sensitivity analysis of different combinations of duration of exercise and carry-over time** | | | |
| --- | --- | --- | --- |
|  | Total SCD events | SCD related to vigorous exertion | Relative risk (95% CI)* |
| **Exercise 30 min, carry-over 60 min** |  |  |  |
| All | 192 | 26 | 31.6 (19.9-50.2) |
| <1 time/week | 161 | 19 | 81.7 (49.2-135.6) |
| 1-2 times/week | 11 | 3 | 27.6 (7.3-104.5) |
| >2 times/week | 20 | 4 | 8.2 (2.7-24.3) |
| **Exercise 30 min, carry-over 0 min** |  |  |  |
| All | 192 | 24 | 87.5 (54.2-141.2) |
| <1 time/week | 161 | 17 | 215.4 (126.1-368.1) |
| 1-2 times/week | 11 | 3 | 83.6 (22.0-318.4) |
| >2 times/week | 20 | 4 | 24.9 (8.35-74.3) |
| **Exercise 60 min, carry-over 0 min** |  |  |  |
| All | 192 | 24 | 43.6 (27.1-70.3) |
| <1 time/week | 161 | 17 | 107.7 (63.4-182.9) |
| 1-2 times/week | 11 | 3 | 41.6 (11.0-157.7) |
| >2 times/week | 20 | 4 | 12.3 (4.1-36.8) |
| **Exercise 60 min, carry-over 30 min** |  |  |  |
| All | 192 | 24 | 29.0 (18.0-46.7) |
| <1 time/week | 161 | 17 | 71.7 (42.3-121.6) |
| 1-2 times/week | 11 | 3 | 27.6 (7.3-104.5) |
| >2 times/week | 20 | 4 | 8.2 (2.7-24.3) |

2) Inclusion of moderate exertion as possible trigger of SCD.

| **Table 4. Relative risk of sudden cardiac death (SCD) within 30 min of moderate to vigorous exertion** | | | |
| --- | --- | --- | --- |
| Frequency of habitual moderate to vigorous exercise | Total SCD events | SCD related to moderate to vigorous exertion | Relative risk (95% CI)* |
| All | 192 | 60 | 129 (88.5-189.2) |
| < 1 time/week | 161 | 49 | 413.9 (277.1-618.2) |
| 1-2 times/week | 11 | 5 | 90.5 (27.4-298.4) |
| >2 times/week | 20 | 6 | 20.4 (7.9-52.6) |
| *Compared to the risk of SCD at other times. CI denotes confidence interval | | | |

3) Excluding cases with an event within 2 years of screening.

| **Table 5. Relative risk of sudden cardiac death (SCD) within 30 min of vigorous exertion** | | | |
| --- | --- | --- | --- |
| Frequency of habitual vigorous exercise | Total SCD events | SCD related to vigorous exertion | Relative risk (95% CI)* |
| All | 165 | 22 | 43.6 (27.1-70.3) |
| < 1 time/week | 138 | 16 | 107.7 (63.4-182.9) |
| 1-2 times/week | 9 | 3 | 41.6 (11.0-157.7) |
| >2 times/week | 18 | 3 | 12.3 (4.1-36.8) |
| *Compared to the risk of SCD at other times. CI denotes confidence interval  Cases with an event within 2 years of screening were excluded. | | | |
